# Unusual Presentation of Necrotizing Enterocolitis Presenting in a Term Infant

**DOI:** 10.7759/cureus.87829

**Published:** 2025-07-13

**Authors:** Erin E Gengo, Sonia Vanegas, Hanna S Sahhar, Jaime Brown

**Affiliations:** 1 Medical School, Edward Via College of Osteopathic Medicine - Carolinas Campus, Spartanburg, USA; 2 Pediatric Intensive Care Unit, Spartanburg Regional Healthcare System, Spartanburg, USA; 3 Pediatrics Department, Spartanburg Regional Healthcare System, Spartanburg, USA

**Keywords:** bloody stool, c-diff, necrotizing enterocolitis (nec), pneumatosis intestinalis, term neonate

## Abstract

Necrotizing enterocolitis (NEC) is a gastrointestinal emergency causing inflammation and necrosis of the intestine, most commonly in premature formula-fed infants. There exists limited conflicting data on the pathophysiology behind NEC development and the underlying intestinal bacteria behind the disease. We report a case of a two-week-old female patient who was born full term. During her birth hospitalization, she was transferred to the neonatal intensive care unit for late-onset tachypnea and apnea, where she received ampicillin and gentamicin intravenously through a peripheral line for a total of 36 hours. The newborn presented to the emergency department (ED) with bloody stool at two weeks of life. Her abdominal X-ray showed possible intramural air with concern for pneumatosis, and the patient was admitted to the pediatric ward with suspected NEC. Stool investigations were positive for occult blood, lactoferrin, and *Clostridioides difficile* (*C. difficile*) DNA amplification but negative for *C. difficile *toxins. The patient was started on intravenous vancomycin, cefepime, and metronidazole as empirical antibiotic coverage. Oral feeds were held for bowel rest. Over the course of the next six days, repeat X-rays were taken daily, and the patient’s symptoms resolved with complete resolution of the X-ray’s abnormalities. The patient was gradually restarted on oral feeds, which were well tolerated by the time of hospital discharge. Our case demonstrates a unique presentation of late-onset NEC in a term infant. This patient case reflects modified Bell criteria stage IIA, including bloody stool with intestinal dilation, ileus, and pneumatosis intestinalis. The concomitant positive test for *C. difficile* in this case could be a causative relationship or just incidental, as it could also be bacterial colonization; therefore, further study is recommended to further investigate the relationship between NEC and* C. difficile* infection (formerly called *Clostridium difficile*), especially in full-term infants.

## Introduction

Necrotizing enterocolitis (NEC) is an inflammatory condition of the bowel, causing the most common surgical emergency in the newborn [1]. Most commonly seen in premature infants, with only 7-15% of all NEC cases happening in term or late preterm infants [2]. Other risk factors for the development of NEC include formula feeding, sepsis, empiric antibiotics, maternal infection, and gastric acid suppression [3].

The pathophysiology of NEC is significantly debated. It is suggested that the increased inflammation occurs due to a combination of genetics, intestinal immaturity, abnormal microbial colonization (although no specific microbial species has been isolated) [2], and a reactive intestinal mucosa [4].

Typically, patients with NEC present with bloody bowel movements and abdominal distension that may be accompanied by symptoms similar to sepsis, such as temperature instability, hypotension, and metabolic acidosis [5]. Common bacterial stool cultures return positive for *Escherichia coli*, *Clostridium perfringens*, and* Klebsiella* [5,6]. The diagnosis of NEC is confirmed via abdominal radiographs (X-ray) visualizing pneumatosis intestinalis and a bubbly appearance with thick-walled, stacked bowel loops [1].

Initial treatment includes withholding enteral feeds and coverage with broad-spectrum antibiotics [5]. With the utilization of Bell’s Criteria [1] for staging, surgical exploratory laparotomy is completed with removal of necrotic bowel [5] when pneumoperitoneum occurs.

This case in particular highlights the importance of needing a further understanding of the pathophysiology behind NEC in a term infant, including intestinal dysbiosis from *Clostridioides difficile *(*C. difficile*) colonization.

## Case presentation

Our two-week-old female patient was born full term at 7 pounds 10.2 ounces (3.464 kilograms). During her initial hospitalization, she was transferred to the neonatal intensive care unit (NICU) for late-onset apnea and tachypnea. A full septic workup was done and revealed negative results. She responded to supplemental oxygen therapy, and a combination of intravenous ampicillin and gentamicin was given for 36 hours. Her symptoms resolved, and she was discharged. 

At two weeks of life, the infant was presented to the emergency department (ED) due to blood found in her stool. Her parents reported the infant had previously been taking her regular feeds of cow's milk-based formula, four ounces every three hours. They reported that they had not changed her formula since birth. The only positive finding on review of systems from the parents was recent sneezing. 

**Figure 1:**
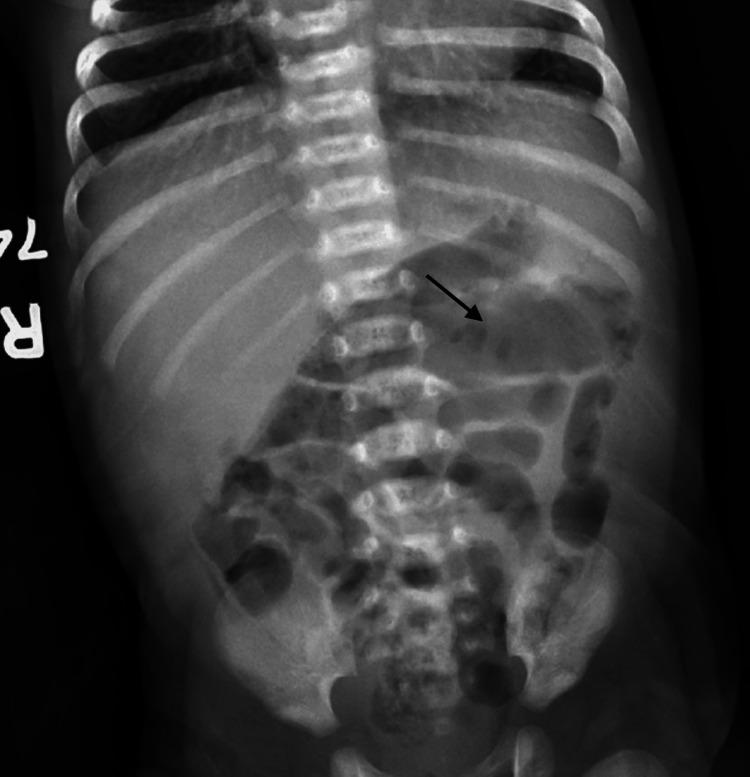
Initial X-ray of the Abdomen with arrow showing possible intramural air in the ascending colon raising concern for pneumatosis, finding that can be associated with necrotizing enterocolitis.

The patient’s vital signs were unremarkable in the ED, with physical examination showing a soft, non-distended abdomen with active bowel sounds in all four quadrants. Laboratory investigations were significant for hemoglobin 15.4 gram per deciliter (g/dL) (normal Range (NR): 12-20 g/dL), platelets 448 x 109 per liter (L) (NR: 150- 450 x109/L), serum bicarbonate 22.3 milliequivalents per liter (mEq/L) (NR: 16 - 25 mEq/L), and C-reactive protein 0.2 milligram per deciliter (mg/dL) (NR: 0.0-0.3 (mg/dL). Stool studies were significant for occult blood and lactoferrin. Initial abdominal X-ray showed possible intramural air in the ascending colon, raising concern for pneumatosis (Figure 1). The patient was admitted and started on intravenous vancomycin, cefepime, and metronidazole with serial abdominal X-rays every six hours. Initial concern for NEC was raised, and a preliminary diagnosis was made. Surgical consultation was done in case escalation of care was needed. 

**Figure 2:**
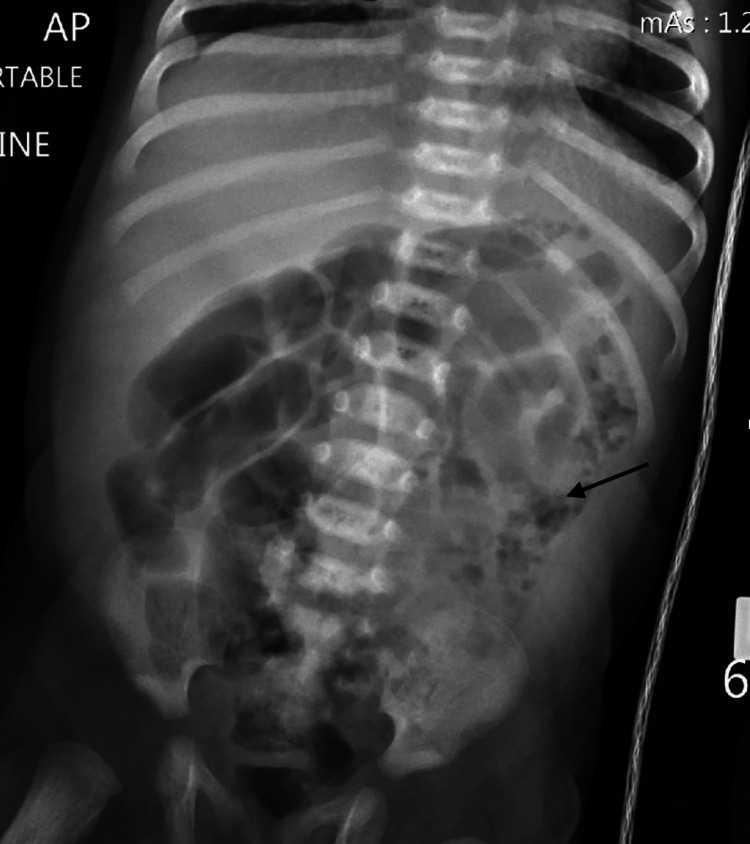
Repeat X-ray with arrow showing bubbly pattern within the splenic flexure and descending colon concerning for necrotizing enterocolitis that is more pronounced than the comparison exam.

The following morning, *C. difficile* DNA amplification was positive, while the toxin antigen test for *C. difficile* was negative. All other bacterial stool toxins, including Salmonella and Shigella, were negative. Repeat abdominal X-ray showed a bubbly air pattern within the splenic flexure that was more pronounced than the comparison exam (Figure 2). The patient was transferred to the pediatric intensive care unit and placed on total parenteral nutrition (TPN) and lipids for bowel rest. Vancomycin was discontinued per the recommendation of the pharmacist due to a lack of toxin production.

**Figure 3:**
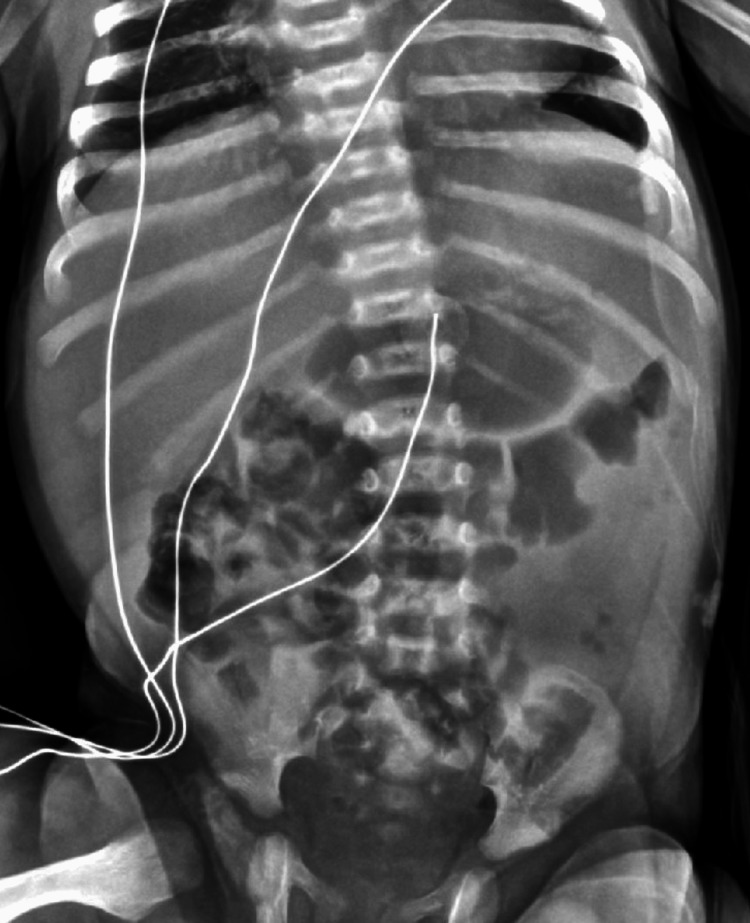
Final X-ray showing normal intestines and complete resolution of the abnormalities.

Over the next six days, X-rays were repeated daily, and the abdomen remained soft and nondistended. Results on the X-rays slowly improved until day eight, when the X-ray showed complete resolution of previously reported abnormalities (Figure 3). The diet was advanced to hypoallergenic infant formula, and the lipids and TPN were discontinued; the patient tolerated these changes well. The patient was discharged home with resolution of symptoms.

## Discussion

According to the literature, NEC occurs more commonly in preterm infants but is relatively rare in term infants, accounting for about 10% of cases. It can be speculated that term infants are less susceptible to NEC, due to a more mature intestinal barrier. However, when NEC is present in this population, it often involves different predisposing factors. This case highlights modified Bell criteria stage IIA NEC in a term infant with concurrent *C. difficile* colonization and a history of a sepsis rule-out with antibiotic exposure, providing insights into the complex interplay of microbial disruption, systemic factors, and NEC pathogenesis.

Unlike preterm infants, whose risk factors for NEC include immaturity of the intestinal barrier, hypoxic-ischemic injury, and early formula feeding, term infants typically have different risk factors such as intrauterine growth retardation (IUGR), birth asphyxia, congenital heart disease, gastroschisis, polycythemia, hypoglycemia, sepsis, exchange transfusion, umbilical lines, milk allergy, premature rupture of membranes with or without chorioamnionitis, and gestational diabetes [6]. Treatment for sepsis-like symptoms, in particular, is an important predisposing factor for NEC during the neonatal period, as it can disrupt intestinal perfusion and alter the gut microbiome. In this case, the patient was treated with ampicillin and gentamicin for 36 hours during her NICU stay for sepsis, which may have contributed to a disruption of the intestinal environment, increasing susceptibility to NEC. The disruption of normal intestinal flora (dysbiosis) is a well-known feature in NEC pathophysiology, characterized by the overgrowth of pathogenic bacteria and a reduction in protective bacteria. There exists a multifactorial mechanism to develop NEC that requires an immature intestinal tract in concurrence with immune-based increase in susceptibility (in this case,* C. difficile *dysbiosis), and an exaggerated chemokine and cytokine host response. In this context, the patient’s prior sepsis rule-out and antibiotic exposure may have played a role in setting the stage for NEC development. The detection of *C. difficile* DNA amplification in this case was not associated with toxin production, which may indicate microbial imbalance rather than direct pathogenicity, as toxin-producing strains are typically associated with active disease [7].

This case shows the importance of having a broad differential diagnosis when a young infant presents with bloody stools, and accounting for all previous exposure to antibiotics and other risk factors for NEC. A thorough workup, including stool studies and imaging, was critical in identifying NEC in this patient. Furthermore, this case shows the potential contribution of microbial dysbiosis in the pathogenesis of NEC in term infants, with C. difficile colonization possibly serving as a marker of an altered gut environment rather than a direct causative agent.

## Conclusions

Although *C. difficile* colonization is common in neonates, its detection in NEC cases can point out the important question about the role of gut microbiota and systemic risk factors. This case demonstrates the need for further research into the interactions between microbial dysbiosis, systemic stressors, and NEC pathogenesis, particularly in term infants. Understanding the relationship could help in creating strategies for prevention and management, such as targeted microbial therapies or approaches to maintain intestinal homeostasis in at-risk populations.
